# Detecting Evolutionary Change-Points with Branch-Specific Substitution Models and Shrinkage Priors

**DOI:** 10.21203/rs.3.rs-6926809/v1

**Published:** 2025-06-25

**Authors:** Xiang Ji, Benjamin Redelings, Shuo Su, Hongcun Bao, Wu-Min Deng, Samuel L. Hong, Guy Baele, Philippe Lemey, Marc A. Suchard

**Affiliations:** 1 Department of Mathematics, School of Science and Engineering, Tulane University, New Orleans, LA, USA; 2 Shanghai Institute of Infectious Disease and Biosecurity, School of Public Health, Fudan University, Shanghai, China; 3 Department of Biochemistry and Molecular Biology, Tulane University, New Orleans, LA, USA; 4 Department of Microbiology, Immunology and Transplantation, Rega Institute, KU Leuven, Leuven, Belgium; 5 Department of Biomathematics, David Geffen School of Medicine, University of California Los Angeles, Los Angeles, CA, USA; 6 Department of Biostatistics, Fielding School of Public Health, University of California Los Angeles, Los Angeles, CA, USA; 7 Department of Human Genetics, David Geffen School of Medicine, University of California Los Angeles, Los Angeles, CA, USA

**Keywords:** linear-time gradient algorithm, branch-specific substitution model, Bayesian inference, maximum likelihood, natural selection

## Abstract

Branch-specific substitution models are popular for detecting evolutionary change-points, such as shifts in selective pressure. However, applying such models typically requires prior knowledge of change-point locations on the phylogeny or faces scalability issues with large data sets. To address both limitations, we integrate branch-specific substitution models with shrinkage priors to automatically identify change-points without prior knowledge, while simultaneously estimating distinct substitution parameters for each branch. To enable tractable inference under this high-dimensional model, we develop an analytical gradient algorithm for the branch-specific substitution parameters where the computation time is linear in the number of parameters. We apply this gradient algorithm to infer selection pressure dynamics in the evolution of the BRCA1 gene in primates and mutational dynamics in viral sequences from the recent mpox epidemic. Our novel algorithm enhances inference efficiency, achieving up to a 90-fold speedup per iteration in maximum-likelihood optimization when compared to central difference numerical gradient method and up to a 360-fold improvement in computational performance within a Bayesian framework using Hamiltonian Monte Carlo sampler compared to conventional univariate random walk sampler.

## 1 Introduction

A striking diversity exists in size, life history, ecology, population structure, physiology, and cellular biology among major groups of organisms. This diversity extends to the genomic level, where substantial interspecies variation in sequence substitution rates and base frequencies is observed. However, standard evolutionary models often assume a homogeneous substitution process across all branches of a phylogenetic tree (Muse and Gaut, 1994; Goldman and Yang, 1994). This assumption is frequently violated in real-world scenarios, particularly in rapidly evolving pathogens like viruses, where evolutionary pressures can vary substantially across lineages due to host shifts, immune escape, drug resistance, or environmental changes (Holmes, 2009; Lemey et al., 2006; Wertheim et al., 2014). The evolution of functional protein-coding genes constitutes another example where potential shifts in evolutionary dynamics could be associated with macroscopic trait changes. Nonsynonymous changes to protein-coding DNA affect the resulting amino acid sequence, whereas synonymous changes do not. Since natural selection mainly operates at the protein level, the ω parameter in the GY94 codon substitution model (Goldman and Yang, 1994) that represents the ratio of nonsynonymous to synonymous codon substitution rates (*dN/dS*) can serve as an informative measure of natural selection. Statistical models that incorporate branch-specific substitution processes offer a flexible and robust framework for detecting such evolutionary changes (Yang and Nielsen, 2002; Huelsenbeck et al., 2000; Pond and Frost, 2005).

Despite their biological relevance, branch-specific models pose substantial statistical challenges, including increased model complexity, risk of overfitting, and the need for efficient computational strategies (Baele et al., 2012, 2016; Höhna et al., 2019). Many studies of lineage-specific variation in selective pressure rely on PAML (Yang, 2007) and use one of two distinct procedures: (1) the user specifies *a priori* clusters of branches on a phylogeny to share a similar *dN/dS* or (2) assumes that each branch has its own *dN/dS*. The first option requires prior knowledge of change-point locations on the tree to group branches to share the same or similar *dN/dS* values. The second option on the other hand is discouraged by the PAML manual as it estimates one parameter for every branch of the tree and tends to be unstable when the tree is large. To address these challenges and enable tractable inference under high-dimensional models, we develop a linear-time analytical gradient algorithm that efficiently computes the derivatives of the log likelihood function with respect to (w.r.t.) branch-specific substitution parameters. This algorithm enables the application of recent advances in Bayesian phylogenetic inference, such as Hamiltonian Monte Carlo (HMC) sampling and shrinkage priors, to facilitate more robust estimation of branch-wise heterogeneity and improve the reliability of these models (Ji et al., 2020; Fisher et al., 2021, 2023). We demonstrate these improvements to infer the selection pressure dynamics in the evolution of the interspecies protein-coding BRCA1 gene in primates (Yang and Nielsen, 2002) and the mutational dynamics in the evolution of mpox virus sequences from a recent outbreak (O’Toole et al., 2023).

## 2 New Approach

In this section, we derive a new analytic gradient algorithm w.r.t. substitution parameters. This new exact gradient algorithm coupled with shrinkage priors and HMC enables scalable Bayesian phylogenetic inference with branch-specific substitution processes without a need to fix the tree topology or prior knowledge of change-points on the tree. The new gradient algorithm leverages the post- and pre-order partial likelihood vectors from Ji et al. (2020) that we briefly review, as well as the numeric implementation in the high-performance BEAGLE library (Ayres et al., 2019; Gangavarapu et al., 2024) for calculating the likelihood and its gradients.

We start by introducing necessary notations and a brief review of the likelihood calculation with post-order traversals. We then derive the *O*(*N*)-dimensional gradient algorithm for substitution parameters (other than the branch lengths) using the post- and pre-order partial likelihood vectors and differential of the instantaneous transition matrices. We demonstrate two specific forms of the gradient w.r.t. substitution parameters of a 4-state nucleotide substitution model and a 61-state codon substitution model. Finally, we review the autocorrelated shrinkage Bayesian bridge prior and HMC methods previously employed to learn evolutionary rates in Fisher et al. (2023) to complete the section.

### 2.1 Notation

We follow the notation defined in Ji et al. (2020) and derive the gradient algorithm. Let ℱ represent a phylogeny with N tips and N-1 internal nodes. We place the root node on the top and the tip nodes at the bottom of ℱ. We denote each node with a number such that 1,2,…,N are for tip nodes and N+1,N+2,…,2N-1 are for internal nodes where the root node is fixed at 2N-1. We denote the parent of node i as pa(i). We refer to the branch (i,pa(i)) by the number i of the child node. On ℱ, we model the columns (i.e., sites) in the sequence alignment as independent and identically distributed such that they arise from conditionally independent continuous-time Markov chains (CTMCs) acting along each branch. More specifically, throughout this manuscript, we consider non-homogeneous CTMCs acting on ℱ where each branch has its own and possibly different CTMC processes.

We denote the state on node i at a site (i.e., single column of the sequence alignment) by $Y_{i}$. To ease the presentation, all our derivation will be for a single site and will omit the summation over sites. Our derivation naturally generalizes to multiple sites with across-site-variation via discretized rate categories (see e.g., Section 2.4; Yang, 1994) or Markov-modulated modeling for competing Markov processes (Baele et al., 2021). We consider a state space of size m (e.g., m=4 for nucleotide substitution models, m=20 for amino acid substitution models and m=61 for codon substitution models that exclude the stop codons). We denote the branch length and the evolutionary rate of branch i by $b_{i}$ and $r_{i}$ respectively, and the real time of node i by $t_{i}$, such that $b_{i}=r_{i}\left(t_{i}-t_{pa(i)}\right)$. For branch i, we denote its infinitesimal rate matrix by $Q_{i}\in R^{m\times m}$ and its eigen decomposition by $Q_{i}=U_{i}\Lambda_{i}U_{i}^{-1}$ such that $U_{i}\in R^{m\times m}$ whose columns are the eigenvectors of $Q_{i}$ and $\Lambda_{i}=diag\left\{\lambda_{i1},\lambda_{i2},\ldots ,\lambda_{im}\right\}$ is a diagonal matrix containing eigenvalues that may be complex. The transition probability matrix $P_{i}$ of branch i becomes $P_{i}=e^{Q_{i}b_{i}}=U_{i}e^{\Lambda_{i}b_{i}}U_{i}^{-1}$. We denote the state distribution at the root node by $\pi ={\left[P\left(Y_{2N-1}=1\right),P\left(Y_{2N-1}=2\right),\ldots ,P\left(Y_{2N-1}=m\right)\right]}^{\prime }$ (not necessarily the stationary distribution of the CTMCs).

We briefly review the post- and pre-order partial likelihood vectors as defined in Ji et al. (2020) and use them to derive the partial derivatives. For any node i on ℱ, we divide the observed characters $Y=\left\{Y_{1},Y_{2},\ldots ,Y_{N}\right\}$ into two disjoint sets and use them to define the corresponding post- and pre-order partial likelihood vectors for the node. We denote the observed characters at the tip nodes that are descendant of node i by $Y_{\left\lfloor i\right\rfloor }$. We denote the complement set of observed characters by $Y_{\left\lceil i\right\rceil }=Y\backslash Y_{\left\lfloor i\right\rfloor }$. We denote the unobserved characters at internal nodes by $y=\left\{Y_{N+1},Y_{N+2},\ldots ,Y_{2N-1}\right\}$. We further denote the branch-specific parameter $\theta_{i}$ that determines branch-specific $Q_{i}$ for branch i and its partial derivative matrix $\partial Q_{i}/\partial \theta_{i}$ such that each entry of $\partial Q_{i}/\partial \theta_{i}$ is a partial derivative of the corresponding entry of $Q_{i}$ w.r.t. $\theta_{i}$. Let $\phi =\left\{ℱ,r_{i},t_{i},\theta_{i},Q_{i};\forall i\right\}$ denote the complete set of all model parameters. Finally, we define the length m post-order partial likelihood vector $p_{i}$ of node i at a site as the conditional probability of observing all data at or below node i (i.e., $Y_{\left\lfloor i\right\rfloor }$) given the state of node i (i.e., $Y_{i}$) such that the sth element of the post-order partial likelihood vector is ${\left(p_{i}\right)}_{s}=P\left(Y_{\left\lfloor i\right\rfloor }|Y_{i}=s\right)$. Similarly, we define the pre-order partial likelihood vector $q_{i}$ of node i as the joint probability of observing the rest of the data (i.e., $Y_{\left\lceil i\right\rceil }$) and the state of node i (i.e., $Y_{i}$) such that the sth element of the pre-order partial likelihood vector is ${\left(q_{i}\right)}_{s}=P\left(Y_{\left\lceil i\right\rceil },Y_{i}=s\right)$. We set the pre-order partial likelihood vector at the root node to be the state distribution at the root node (i.e., $q_{2N-1}=\pi$).

### 2.2 Likelihood

If the states of the internal nodes are all observed, one could write out the joint likelihood by

(1) $$P\left(Y,y\right)=P\left(Y_{2N-1}\right)\prod_{j=1}^{2N-2}P\left(Y_{j}|Y_{pa\left(j\right)}\right).$$

However, the data likelihood is the probability of the observed discrete characters at the tip nodes by marginalizing over all possible latent states at the internal nodes

(2) $$P\left(Y\right)=\sum_{Y_{N+1}}\sum_{Y_{N+2}}\ldots \sum_{Y_{2N-1}}P\left(Y,y\right).$$

We omit the conditioning on the parameters ϕ above and in later derivations for ease of notation. Ji et al. (2020) derived the calculations of the post- and pre-order partial likelihood vectors. We summarize the updates of the partial likelihood vectors here with more detailed calculations of the transition probability matrix to later apply them for the calculation of the derivatives w.r.t. substitution parameters. We initialize the post-order partial likelihood vectors at the tip nodes by the corresponding observed sequence state at a site such that $P\left(Y_{\left\lfloor i\right\rfloor }|Y_{i}=s\right)=P\left(Y_{i}|Y_{i}=s\right)=1_{\left\{Y_{i}=s\right\}}$ for i=1,2,…,N,j=1,2,…,m. One can modify the post-order partial likelihood vector to account for partially observed and missing data at the tip node (Felsenstein, 1981). The updates of post-order partial likelihood vectors occur through a post-order traversal where descendant nodes on the phylogenetic tree are visited before their parent nodes. More specifically, the update of the post-order partial likelihood vector $p_{k}$ at internal node k with two descendant nodes i and j (i.e., pa(i)=pa(j)=k) where $p_{i}$ and $p_{j}$ are available falls out as

(3) $$p_{k}=P_{i}p_{i}\circ P_{j}p_{j}.$$

After finishing the post-order traversal, the data likelihood is

(4) $$P\left(Y\right)=p_{2N-1}^{\prime }\pi .$$

Similarly, we initialize the pre-order partial likelihood vector at the root node by the state distribution at the root (i.e., $q_{2N-1}=\pi$) and update the pre-order partial likelihood vectors through a pre-order traversal that visits nodes on the phylogenetic tree in a parent-node-first manner in the reverse order of a post-order traversal. This way, the update of the pre-order partial likelihood vector $q_{i}$ at internal node i with its sister node j and parent node k becomes

(5) $$q_{i}=P_{i}^{\prime }\left[q_{k}\circ \left(P_{j}p_{j}\right)\right],$$

where transition probability matrices $P_{i}$ and $P_{j}$ are available in the post-order traversal when calculating the likelihood. With the pre- and post-order partial likelihood vectors, one can rewrite Equation 2 by marginalizing over the states of any single node such that

(6) $$\begin{array}{ll} P(Y) & =\sum_{Y_{i}}P\left(Y,Y_{i}\right)\\ & =\sum_{Y_{i}}P\left(Y_{\left\lfloor i\right\rfloor }|Y_{i}\right)P\left(Y_{\left\lceil i\right\rceil },Y_{i}\right)\\ & =p_{i}^{\prime }q_{i}. \end{array}$$

Equation 6 forms the basis for the gradient algorithm and holds for all nodes (i.e., i=1,2,…,2N-1). The likelihood calculation at the root after finishing the post-order traversal (i.e., Equation 4) is a special case for i=2N-1.

**Figure 1:**
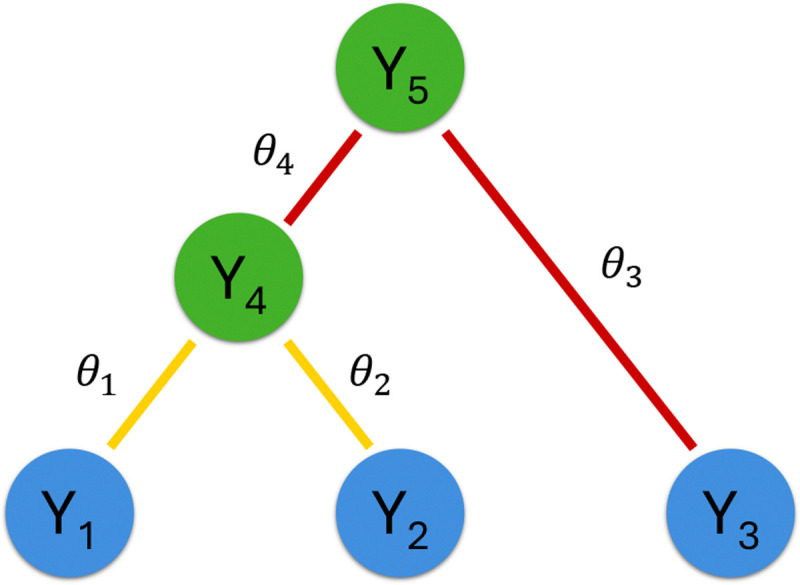
Example of a 3-taxon tree. Sequence states $Y={\left(Y_{1},Y_{2},Y_{3}\right)}^{\prime }$ are observed data at the tips of the tree. The latent states $Y_{4}$ and $Y_{5}$ are unobserved at internal nodes of the tree. For node 4, its post-order partial likelihood vector contains the conditional probability of $P\left(Y_{\left\lfloor 4\right\rfloor }|Y_{4}\right)=P\left(Y_{1},Y_{2}|Y_{4}\right)$ and its pre-order partial likelihood vector contains the joint probability of $P\left(Y_{\left\lceil 4\right\rceil },Y_{4}\right)=P\left(Y_{3},Y_{4}\right)$. We further color the branches to show the update of the two partial likelihood vectors at internal node 4 such that yellow branches correspond to the update of the post-order partial likelihood vectors and red branches correspond to the update of the pre-order partial likelihood vectors.

Figure 1 illustrates these quantities using a 3-taxon tree example. The observed data in Figure 1 are $Y=\left\{Y_{1},Y_{2},Y_{3}\right\}$. One obtains the likelihood of the observed data by marginalizing over $y=\left\{Y_{4},Y_{5}\right\}$. Two possible post-order traversals for the example tree in Figure 1 are 1→2→4→3→5 and 1→2→3→4→5. The reverse of these post-order traversals form the pre-order traversals. In the post-order traversal, one calculates the transition probability matrices at branches 1 to 4 by $P_{i}=U_{i}e^{\Lambda_{i}b_{i}}U_{i}^{-1},\;i=1,2,3,4$ where the eigen decomposition is either analytically (e.g., Tamura and Nei (1993) model and its nested special cases) or numerically available. The updates of post-order partial likelihood vectors become $p_{4}=P_{1}p_{1}\circ P_{2}p_{2}$ and $p_{5}=P_{4}p_{4}\circ P_{3}p_{3}$ where post-order partial likelihood vectors ($p_{1},\;p_{2},\;p_{3}$) are formed according to the observed sequence state at the corresponding tip nodes. For the pre-order traversal, one initializes the pre-order partial likelihood vector at the root node by setting $q_{5}=\pi$. The updates of pre-order partial likelihood vectors become $q_{4}=P_{4}^{\prime }\left[q_{5}\circ \left(P_{3}p_{3}\right)\right],\;q_{3}=P_{3}^{\prime }\left[q_{5}\circ \left(P_{4}p_{4}\right)\right],\;q_{2}=P_{2}^{\prime }\left[q_{4}\circ \left(P_{1}p_{1}\right)\right]$ and $q_{1}=P_{1}^{\prime }\left[q_{4}\circ \left(P_{2}p_{2}\right)\right]$.

### 2.3 Gradient

Ji et al. (2020) detail the calculation of the derivative w.r.t. branch lengths, hence we will here focus on the calculation of the derivative w.r.t. parameters within the infinitesimal generator matrix. With the likelihood expanded at node i as in Equation 6, we derive the corresponding scalar component of gradient vector which is the first order derivative w.r.t. parameter $\theta_{i}$ (i.e., $\left[\nabla log\;P(Y){]}_{i}=\frac{\partial }{\partial \theta_{i}}log\;P(Y\right)$). To do this, we adopt a “spectral representations” approach to calculate the first directional derivative of the matrix exponential method as in Najfeld and Havel (1995). We first derive the derivative of the transition probability matrix $P_{i}$ w.r.t. $\theta_{i}$,

(7) $$\begin{array}{ll} \frac{\partial P_{i}}{\partial \theta_{i}} & \;=\frac{\partial e^{Q_{i}b_{i}}}{\partial \theta_{i}}\\ & =U_{i}\left[{\bar{Q}}_{i}\circ \Phi_{i}\left(b_{i}\right)\right]U_{i}^{-1}, \end{array}$$

where ${\bar{Q}}_{i}=U_{i}^{-1}\frac{\partial Q_{i}}{\partial \theta_{i}}U_{i}$ and ∘ denotes the element-wise product. The matrix $\Phi_{i}\left(b_{i}\right)$ is symmetric with entries

(8) $${\left\{\Phi_{i}\left(b_{i}\right)\right\}}_{jk}={\left\{\Phi_{i}\left(b_{i}\right)\right\}}_{kj}=\left\{\begin{array}{ll} \left(e^{b_{i}\lambda_{ij}}-e^{b_{i}\lambda_{ik}}\right)/\left(\lambda_{ij}-\lambda_{ik}\right) & \text{if}\;\lambda_{ij}\neq \lambda_{ik}\\ b_{i}e^{b_{i}\lambda_{ij}} & \text{if}\;\lambda_{ij}=\lambda_{ik}, \end{array}\right.$$

and the derivative of the pre-order partial likelihood vector $q_{i}$ w.r.t. $\theta_{i}$ becomes

(9) $$\begin{array}{ll} \frac{\partial q_{i}}{\partial \theta_{i}} & =\frac{\partial }{\partial \theta_{i}}\left\{P_{i}^{\prime }\left[q_{k}\circ \left(P_{j}p_{j}\right)\right]\right\}={\left(\frac{\partial }{\partial \theta_{i}}e^{Q_{i}b_{i}}\right)}^{\prime }\left[q_{k}\circ \left(P_{j}p_{j}\right)\right]\\ & ={\left\{e^{Q_{i}b_{i}}e^{-Q_{i}b_{i}}\cdot U_{i}\left[{\bar{Q}}_{i}\circ \Phi_{i}\left(b_{i}\right)\right]U_{i}^{-1}\right\}}^{\prime }\left[q_{k}\circ \left(P_{j}p_{j}\right)\right]\\ & ={\left\{e^{Q_{i}b_{i}}\left[U_{i}e^{-\Lambda_{i}b_{i}}U_{i}^{-1}\right]\cdot U_{i}\left[{\bar{Q}}_{i}\circ \Phi_{i}\left(b_{i}\right)\right]U_{i}^{-1}\right\}}^{\prime }\left[q_{k}\circ \left(P_{j}p_{j}\right)\right]\\ & ={\left\{U_{i}\left[{\bar{Q}}_{i}\circ \left(e^{-\Lambda_{i}b_{i}}\Phi_{i}\left(b_{i}\right)\right)\right]U_{i}^{-1}\right\}}^{\prime }\left\{P_{i}^{\prime }\left[q_{k}\circ \left(P_{j}p_{j}\right)\right]\right\},\\ & ={\left\{U_{i}\left[{\bar{Q}}_{i}\circ \Psi_{i}\left(b_{i}\right)\right]U_{i}^{-1}\right\}}^{\prime }q_{i}, \end{array}$$

where matrix $\Psi_{i}\left(b_{i}\right)=e^{-\Lambda b_{i}}\Phi_{i}\left(b_{i}\right)$ is symmetric with entries

(10) $${\left\{\Psi_{i}\left(b_{i}\right)\right\}}_{jk}={\left\{\Psi_{i}\left(b_{i}\right)\right\}}_{kj}=\left\{\begin{array}{ll} \left[1-e^{b_{i}\left(\lambda_{ik}-\lambda_{ij}\right)}\right]/\left(\lambda_{ij}-\lambda_{ik}\right) & \text{if}\;\lambda_{ij}\neq \lambda_{ik}\\ b_{i} & \text{if}\;\lambda_{ij}=\lambda_{ik}. \end{array}\right.$$

Finally, the derivative of the log likelihood w.r.t. $\theta_{i}$ falls out as

(11) $$\begin{array}{ll} \frac{\partial }{\partial \theta_{i}}log\;P(Y) & =\frac{\partial }{\partial \theta_{i}}\left[p_{i}^{\prime }q_{i}\right]/P(Y)\\ & =p_{i}^{\prime }\frac{\partial q_{i}}{\partial b_{i}}/P(Y)\\ & =q_{i}^{\prime }\left\{U_{i}\left[{\bar{Q}}_{i}\circ \Psi_{i}\left(b_{i}\right)\right]U_{i}^{-1}\right\}p_{i}/P(Y). \end{array}$$

### 2.4 Likelihood and gradient with substitution rate heterogeneity

Equation 11 does not consider substitution rate heterogeneity across sites. A popular approach to model the substitution rate heterogeneity across sites is by using a hidden Markov model (HMM) where the substitution rate of a site belongs to one of multiple rate categories which form discrete hidden states (Yang, 1994). For discrete rate category l with rate $\gamma_{l}$, the transition probability matrix for branch k of rate category l becomes $P_{k|\gamma_{l}}=e^{Q_{k}b_{k}\gamma_{l}}$. As in HMMs, the observed data likelihood marginalizes over all possible hidden states and becomes the weighted sum of the conditional likelihood of each rate category:

(12) $$\begin{array}{rr} P(Y) & =\sum_{\gamma_{l}}P\left(Y|\gamma_{l}\right)P\left(\gamma_{l}\right)\\ & =\sum_{\gamma_{l}}p_{k|\gamma_{l}}^{\prime }q_{k|\gamma_{l}}P\left(\gamma_{l}\right), \end{array}$$

where $p_{k|\gamma_{l}}$ and $q_{k|\gamma_{l}}$ are the corresponding post- and pre-order partial likelihood vectors at node k for rate category l. Ji et al. (2020) detailed their updates. Similarly, the numerator and denominator of Equation 11 become weighted sums in the rate heterogeneous case:

(13) $$\frac{\partial }{\partial b_{i}}log\;P(Y)=\sum_{\gamma_{l}}\gamma_{l}q_{i|\gamma_{l}}^{\prime }\left\{U_{i}\left[{\bar{Q}}_{i}\circ \Psi_{i}\left(b_{i}\gamma_{l}\right)\right]U_{i}^{-1}\right\}p_{i|\gamma_{l}}P\left(\gamma_{l}\right)/P(Y).$$

Similar to the calculation of the gradient w.r.t. branch lengths (Ji et al., 2020), Equation 9 and Equation 13 show that we can reuse the post- and pre-order partial likelihood vectors $p_{i},\;q_{i}$ by constructing a modified partial derivative matrix ($U_{i}\left[{\bar{Q}}_{i}\circ \Psi_{i}\left(b_{i}\right)\right]U_{i}^{-1}$ for rate homogeneity and $U_{i}\left[{\bar{Q}}_{i}\circ \Psi_{i}\left(b_{i}\gamma_{l}\right)\right]U_{i}^{-1}$ for rate heterogeneity across sites) at node i for calculating the partial derivative of branch i. Since the modified partial derivative matrix construction is only branch-dependent, we can calculate these branch-specific derivatives together with the update of the pre-order partial likelihood vectors. The derivative w.r.t. different substitution parameters is associated with different partial differential matrices and their calculation can happen at the same time using the same set of post- and pre-order partial likelihood vectors. This procedure gives us the gradient vector of all partial derivatives w.r.t. branch 1,2,…,2N-2 in one single pre-order traversal in linear-time.

### 2.5 Example analytic derivatives w.r.t. substitution parameters

We explore two specific branch-specific substitution models to learn the evolutionary dynamics of selection pressure and mutational changes. We refer to the 4-state nucleotide substitution model as the HKY+APOBEC model that extends the HKY model (Hasegawa et al., 1985) to account for excess C to T and G to A transitions due to effect of host APOBEC proteins in mpox evolution (O’Toole et al., 2023). The other is a 61-state codon substitution model denoted as F1×4MG+κ+ω by the codeml software (Yang, 2007) with branch-specific ω parameters. We derive the analytic derivative forms w.r.t. APOBEC-effect parameter τ and the *dN/dS* parameter ω of these two substitution models respectively using Equation 11. For simplicity, we drop the subscript i and derive the derivative form without rate heterogeneity that is the equivalent form for one single rate category when considering rate heterogeneity.

The HKY + APOBEC model has the off-diagonal instantaneous rate $Q_{ij}$ from nucleotide i to j be

(14) $$Q_{ij}=\left\{\begin{array}{ll} \pi_{j} & \text{for}\;\text{a}\;\text{transversion}\\ \pi_{j}\kappa & \text{for}\;\text{a}\;\text{non-APOBEC}\;\text{transition}\\ \pi_{j}\kappa \tau & \text{for}\;\text{an}\;\text{APOBEC}\;\text{transition}, \end{array}\right.$$

where APOBEC transitions correspond to (i,j)=(C,T) and (i,j)=(G,A) (see Section 3.1 for more details) and the APOBEC-parameter τ>0 captures the multiplicative effect such that τ>1 indicates excess APOBEC transition rates. The rows of Q sum to 0 so that the diagonal entries of Q are the negative of the row sum of off-diagonal entries $\left(Q_{ii}=-\sum_{j\neq i}Q_{ij}\right)$. The off-diagonal entry of differential matrix ∂Q/∂θ is then

(15) $${\left(\frac{\partial Q}{\partial \theta }\right)}_{ij}=\left\{\begin{array}{ll} \pi_{j}\kappa & \text{for}\;\text{an}\;\text{APOBEC}\;\text{transition}\\ 0 & \text{otherwise,} \end{array}\right.$$

where row sums of ∂Q/∂θ are 0.

The F1×4MG+κ+ω codon substitution model has the instantaneous rate $Q_{ij}$ from codon triplet i to j be 0 if i and j differ in more than one of their three positions. If i and j differ in exactly one nucleotide that has type h(h∈{A,C,G,T}) in codon j, the instantaneous rate becomes

(16) $$Q_{ij}=\left\{\begin{array}{ll} \pi_{h} & \text{for}\;\text{a}\;\text{synonymous}\;\text{transversion}\\ \pi_{h}\kappa & \text{for}\;\text{a}\;\text{synonymous}\;\text{transition}\\ \pi_{h}\omega & \text{for}\;\text{a}\;\text{nonsynonymous}\;\text{transversion}\\ \pi_{h}\kappa \omega & \text{for}\;\text{a}\;\text{nonsynonymous}\;\text{transition}, \end{array}\right.$$

and the differential matrix ∂Q/∂θ has off-diagonal entries

(17) $${\left(\frac{\partial Q}{\partial \theta }\right)}_{ij}=\left\{\begin{array}{ll} \pi_{h} & \text{for}\;\text{a}\;\text{nonsynonymous}\;\text{transversion}\\ \pi_{h}\kappa & \text{for}\;\text{a}\;\text{nonsynonymous}\;\text{transition}\\ 0 & \text{otherwise.} \end{array}\right.$$

Similar to the case of the HKY + APOBEC model, Q and ∂Q/∂θ of the F1×4MG+κ+ω model have row sums of 0. While Equations 15 and 17 only form the differential matrix, one can easily construct the Ψ matrix through Equation 10 and $\bar{Q}$ using the eigen decomposition of the instantaneous transition matrices Q.

### 2.6 Autocorrelated shrinkage branch-specific substitution parameters

We assume the branch-specific substitution parameters $\theta =\left\{\theta_{1},\ldots ,\theta_{2N-2}\right\}$ are autocorrelated. Similar to the treatment of branch-specific evolutionary rates in Fisher et al. (2023), we model the autocorrelation in terms of the incremental difference $\phi_{i}$ between the log-transformed values of branch i’s substitution parameter and its parent lineage’s substitution parameter:

(18) $$\phi_{i}=log\;\theta_{i}-log\;\theta_{pa(i)},\;\text{for}\;i\in \{1,\ldots ,2N-2\}\;\text{and}\;\text{let}\;\theta_{2N-1}\equiv 1.$$

Under this parameterization, the increments $\phi =\left\{\phi_{1},\ldots ,\phi_{2N-2}\right\}\in R^{2N-2}$ are a linear transformation of logθ. To shrink the total number of substitution parameter changes along the tree, we employ shrinkage priors on $\phi_{i}$’s such that $\phi_{i}\overset{iid}{∼}P_{\phi }$ and $E\left(\phi_{i}\right)=0$. Typically, $P_{\phi }$ may follow a Gaussian (Thorne et al., 1998), Laplace or Horseshoe distribution (Carvalho et al., 2010). We choose the flexible, heavy-tailed, Bayesian bridge prior (Polson et al., 2014) on the increments,

(19) $$P_{\phi }\propto \exp\left\{-{\left|\frac{\phi_{i}}{\mu }\right|}^{\alpha }\right\},$$

where μ>0 is termed the “global scale” and α∈(0,1] changes the shape of $P_{\phi }$ so that smaller α places more mass near zero. When $\alpha =1,\;P_{\phi }$ is the Laplace prior. When α is closer to 0, the Bayesian bridge prior approaches the best subset selection when used in a regression setting. In both examples, we set α=0.9 to enforce slightly weaker shrinkage compared to Fisher et al. (2023). Therefore, the joint prior for all increments is the product $p(\phi )\propto \prod_{i=1}^{2N-2}exp\left\{-{\left|\frac{\phi_{i}}{\mu }\right|}^{\alpha }\right\}$.

### 2.7 Hamiltonian Monte Carlo method

We briefly review the HMC sampling method. HMC has demonstrated effective performance in learning high-dimensional posterior distributions associated with modern phylodynamic models (Ji et al., 2020; Fisher et al., 2021, 2023; Ji et al., 2023; Hassler et al., 2023). The linear-time analytical gradient algorithm developed in this manuscript further expands application of HMC. HMC is a state-of-the-art Markov chain Monte Carlo (MCMC) method that proposes new values for all parameters with relatively high acceptance rate by exploiting Hamiltonian dynamics (Neal, 2011). To sample from the posterior distribution π(θ), HMC introduces an auxiliary momentum parameter p usually drawn from a multivariate normal distribution with mean 0 and variance-covariance matrix M that is also referred to as the ‘mass matrix’ (i.e., p∼𝒩(0,M)). The auxiliary momentum parameter has ‘kinetic energy’ of $K(p)=p^{\prime }M^{-1}p/2$ (due to the multivariate normal kernel). HMC treats the negative log density of π(θ) as the ‘potential energy’ U(θ)=-log(π(θ)) and the combination of the potential and kinetic energies form the Hamiltonian function H(θ,p)=U(θ)+K(p). HMC generates a Metropolis proposal (Metropolis et al., 1953) by simulating Hamiltonian dynamics from the current state ($\theta_{0},\;p_{0}$) for a pre-defined integration time according to the differential equation:

(20) $$\begin{array}{l} \frac{d\theta }{\;dt}=\nabla K(p)=M^{-1}p\\ \frac{dp}{\;dt}=-\nabla U(\theta )=\nabla log\;\pi (\theta ), \end{array}$$

that is often numerically integrated through the *leapfrog* method (Neal, 2011) as an approximation. The leapfrog method performs n step discrete integration with a step size of ϵ such that the total integration time becomes nϵ. For each leapfrog step, one iteratively updates the momentum and position by

(21) $$\begin{array}{ll} p_{t+\epsilon /2} & =p_{t}+\frac{\epsilon }{2}\nabla \;log\;\pi \left(\theta_{t}\right)\\ \theta_{t+\epsilon } & =\theta_{t}+\epsilon M^{-1}p_{t+\epsilon /2}\\ p_{t+\epsilon } & =p_{t+\epsilon /2}+\frac{\epsilon }{2}\nabla \log\pi \left(\theta_{t+\epsilon }\right). \end{array}$$

The leapfrog integration starts from time t=0 and ends at t=nϵ where the ending position $\theta_{n\epsilon }$ is the proposed state. The proposed state is then accepted with probability

(22) $$min\left\{1,exp\left[H\left(\theta_{0},p_{0}\right)-H\left(\theta_{n\epsilon },p_{n\epsilon }\right)\right]\right\},$$

that depends on how well the numerical integration preserves the Hamiltonian energy.

The geometric structure of the posterior distribution substantially influences the computational efficiency of HMC. Specifically, when individual parameters exhibit varying scales within the posterior, neglecting this structural variation can lead to a significant reduction in HMC efficiency (Neal, 2011; Ji et al., 2020; Fisher et al., 2023; Ji et al., 2023). To account for structural variability in the posterior, we adopt the preconditioning mass matrix informed by the absolute value of the diagonal Hessian of the log-prior density as developed in Fisher et al. (2023). The diagonal mass matrix weights momentum draws by prior increment precision.

## 3 Materials and Methods

### 3.1 Sequence data sets

We examine the mutational dynamics in the molecular evolution of the 2022 mpox epidemic in North America (O’Toole et al., 2023; Paredes et al., 2024) and the classic tumor suppressor BRCA1 genes from primates (Yang and Nielsen, 2002).

#### MPXV

Mpox is a viral zoonotic disease caused by the mpox virus (MPXV) endemic to West and Central Africa. MPXV is a double-stranded DNA virus and was first discovered in 1958 in Copenhagen (Cho and Wenner, 1973). In July 2022, the World Health Organization declared mpox a public health emergency of international concern. The DNA sequences for the first cases from the 2022 mpox epidemic shared 42 nucleotide differences from the closest MPXV sequences sampled prior to the outbreak. Almost all of these mutations are typical of the activity of APOBEC3 deaminases, which are host enzymes that play a role in antiviral defense. Assuming APOBEC3 editing occurs at a higher rate during human MPXV infection than in the previous host, O’Toole et al. (2023) developed a dual-process molecular clock model to account for possible elevated APOBEC3 mutation rates and estimated that MPXV had been circulating in humans since 2016. Interestingly, most of the observed nucleotide changes associated with the MPXV sequences from the 2022 epidemic appear to be a particular dinucleotide change from TC → TT and its reverse complement, GA → AA. In light of this specific dinucleotide mutation pattern, we extend the HKY model with a new parameter to account for possible excess C → T and G → A mutations as illustrated in Equation 14. We estimate the dynamic of APOBEC3 mutation rates in recent MPXV evolution. The MPXV data example consists of 138 genomes with an alignment of 197,209 nucleotides.

#### BRCA1 gene

The BRCA1 tumor suppressor gene contributes to preserving genomic integrity by participating in recombinational and transcription-coupled DNA repair, as well as regulating transcription. Mutations in the BRCA1 gene are associated with a higher risk of developing breast cancer in women. In their landmark study, Yang and Nielsen (2002) studied the protein coding sequences of BRCA1 in primates using a branch-specific ω model and observed an elevated estimate of *dN/dS* values (i.e., the ω parameter) in the clade of human and chimpanzee that suggested positive selection. We re-examine this classic data set that contains 7 ingroup primate species (human, chimpanzee, gorilla, orangutan, macaca, howler monkey and bushbaby) and an outgroup species (flying lemur) with an alignment of 1,123 codons.

### 3.2 Other models and priors

We employ branch-specific substitution processes as detailed in Section 2.5 in our analyses. We place Bayesian bridge shrinkage priors on the log difference of the branch-specific substitution parameter of a branch and that of its parent branch with the global scale μ=1 and exponent α=0.9 for both analyses. We pick this specific exponent value to shrink the total number of change-points in the posterior across the tree to around 2 for the MPXV data example and use the same exponent value for the BRCA1 example. We place a normal prior with mean 0 and variance 1 on the log of the kappa parameter in the HKY model for the MPXV analysis and an exponential prior with mean 1 on the kappa parameter for the BRCA1 analysis. We place a coalescent prior with exponentially growing effective population size on ingroup taxa and constant effective population size on outgroup taxa on the tree topology for the MPXV analysis (please see the BEAST XML files for details of ingroup and outgroup taxa) and a fixed species tree topology for the BRCA1 analysis. We employ a random-effects molecular clock model as described in Ji et al. (2020) for the MPXV analysis and a fixed evolutionary rate of 1 for the BRCA1 analysis. We employ a discretized gamma distribution with 4 rate categories for modeling among-site rate heterogeneity (Yang, 1994) for both analyses and place an exponential prior with mean 0.5 on its shape parameter.

### 3.3 Implementation

The gradient algorithm derived in Equation 9 and Equation 13 uses the same BEAGLE (Ayres et al., 2019) infrastructure with a CPU implementation as described in Ji et al. (2020) and a multi-core GPU implementation as in Gangavarapu et al. (2024). We implement the construction of the modified partial differential matrix within the development branch “hmc-clock” of BEAST X (Suchard et al., 2018; Baele et al., 2025) for the demonstrations in this paper. We provide instructions, commit tags of BEAGLE and BEAST X software packages, and the BEAST XML files for reproducing these analyses on GitHub at https://github.com/suchard-group/BranchSpecificSupplementary.

## 4 Results

We demonstrate the computational efficiency gains of our linear-time gradient algorithm for inferring the branch-specific substitution parameters using two empirical data examples of different sizes. The first dataset (MPXV) includes a large sample of 138 complete genomes, while the second (BRCA1) includes a small sample of 8 sequences. For each dataset, we present results from both optimization (via L-BGFS) and Bayesian posterior sampling (via MCMC). We compare performance of the analytic gradients versus central-difference numerical gradients by applying them separately in the gradient-based L-BFGS optimization routine (see e.g., Dennis Jr and Schnabel (1996)). For posterior sampling, we assess the efficiency of HMC relative to single-variable Metropolis-Hastings proposals.

### 4.1 Optimization

We obtain the maximum likelihood estimate (MLE) of the branch-specific substitution parameters conditional on all other parameters via the L-BFGS optimization algorithm for both datasets. We compare the performance of our analytic gradient method with an often-used central finite difference numerical scheme. The numerical scheme calculates the derivative of one branch-specific substitution parameter via two likelihood evaluations and therefore has a complexity of *O*(*N*^2^) for the gradient w.r.t. all *O*(*N*) dimensions. On the other hand, our analytic approach scales linearly w.r.t. number of sequences and only requires *O*(*N*) computation to achieve the same gradient. Table 1 provides a summary of the comparison, highlighting the significant performance improvement of our analytic method across the two datasets. In terms of overall computational time for the MLE estimation procedure, the analytic gradient method achieves a performance advantage, offering approximately a 271.7-fold speedup for the MPXV example and a 1.4-fold speedup for the BRCA1 example compared to the central finite difference numerical gradient method. When averaged over each iteration of the MLE estimation procedure, the analytic gradient method achieves an 89.9-fold speedup for the MPXV example and a 4.3-fold speedup for the BRCA1 example compared to the central finite difference numerical gradient method. The analytic gradient also achieves higher ending maximum log likelihood values in both examples.

### 4.2 Posterior inference

#### MPXV

**Figure 2:**
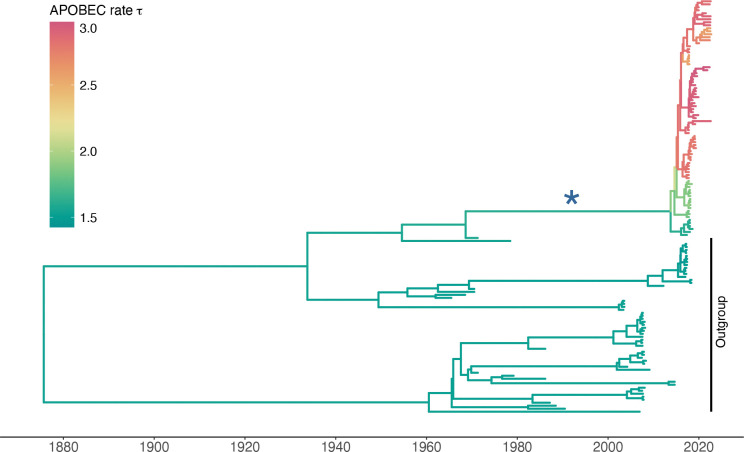
Maximum clade credibility tree of the MPXV analysis. The dataset consists of 138 sequences of the mpox virus. Branches are color-coded by the posterior means of the branch-specific APOBEC rate τ. The blue star indicates the branch after which the acceleration of APOBEC effect started.

We infer the posterior distribution of all parameters of the model. For the MPXV analysis, this includes the substitution parameters, internal node heights, tree topology, shape of the discrete gamma rate heterogeneity model, and evolutionary rates. We run two independent MCMC analyses with different random seed numbers to ensure convergence in BEAST X (Suchard et al., 2018; Baele et al., 2025) using BEAGLE (Ayres et al., 2019; Gangavarapu et al., 2024) for parallel computation using GPU (for the full analysis) and CPU with Streaming SIMD Extensions (SSE; for benchmarking). Our analysis estimates the tree-wise (fixed-effect) mean rate with posterior mean 1.24 (95% Bayesian credible interval: 0.93, 1.59) ×10^−5^ substitutions per site per year and an estimated variability characterized by the scale parameter of the lognormal distributed branch-specific random-effects with posterior mean 1.80 (1.26, 2.41). Our estimated evolutionary rate (over the entire sequence) is in between the estimated values of APOBEC3 and non-APOBEC3 evolutionary rates by O’Toole et al. (2023). Figure 2 shows the maximum clade credibility tree of the MPXV example. Our analysis revealed a clear acceleration of the APOBEC effect in the ingroup samples after the split indicated by the blue star branch. The estimated time of the split has 95% posterior estimate of 2012.3 (2010.8, 2016.0).

#### BRCA1

**Figure 3:**
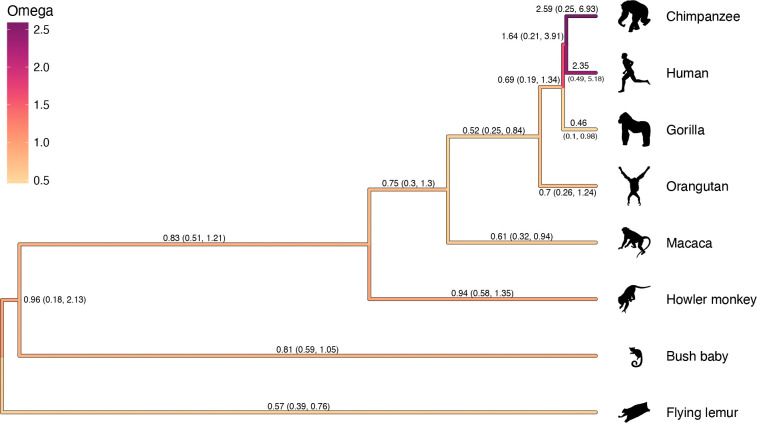
Maximum clade credibilty tree of the BRCA1 analysis. Numbers on branches represent the posterior mean estimates for the branch-specific ω parameter with numbers in the parenthesis showing the 95% Bayesian credible intervals. Branches are color-coded according to the posterior means of the branch-specific ω parameter. Taxon silhouettes were sourced from PhyloPic ( phylopic.org); see link for attribution.

We fix the tree topology and infer a rooted tree without imposing any molecular clock models for the BRCA1 analysis such that branch lengths are in the unit of expected codon substitutions per site (i.e., equivalent as inferring a time-calibrated tree with fixed molecular clock rate of 1). Our analysis estimates the transition to transversion ratio parameter κ with posterior mean 4.6 (4.0, 5.3). Figure 3 shows the estimated posterior means of the branch-specific *dN/dS* parameter ω on each branch. Our analysis reaffirms the elevated values of the *dN/dS* ratios leading to the clade of human and chimpanzee as originally revealed by Yang and Nielsen (2002) while allowing each branch to have its own parameter without any prior allocations on change-point locations.

### 4.3 Sampling efficiency

**Figure 4:**
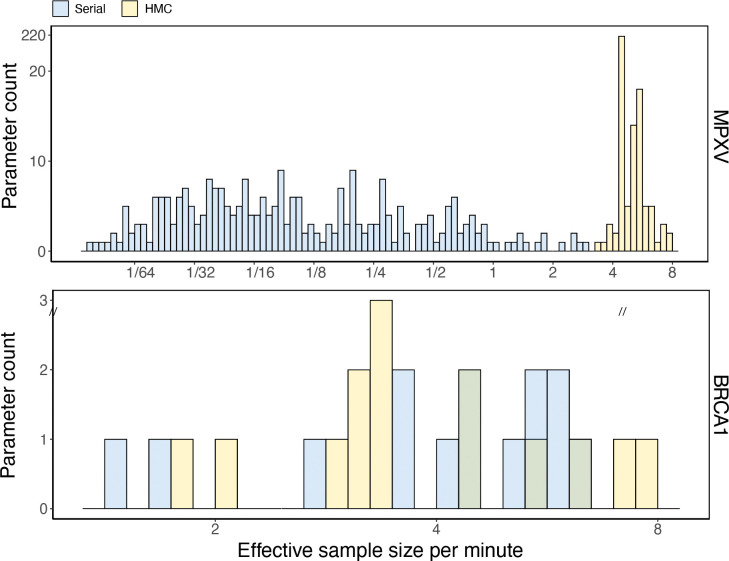
Posterior sampling efficiency on all branch-specific substitution parameters for the BRCA1 and MPXV examples. We bin parameters by their log_2_(ESS/min) values. The univariate and the HMC transition kernels employed in the MCMC are color-coded.

We infer the conditional posterior distribution of all branch-specific substitution parameters using an HMC and a univariate Metropolis-Hastings transition kernel for the MPXV and BRCA1 examples. We refer to the univariate transition kernel as ‘univariate’. The ‘univariate’ updates propose new values for a single dimension in the high-dimensional branch-specific substitution parameter at a time where the HMC updates propose new values for all dimensions simultaneously by integrating the Hamiltonian dynamics (see Section 2.7). We compare the efficiency of these two transition kernels by their ability to accumulate effective sample size (ESS) per unit time for estimating the high-dimensional branch-specific substitution parameters. For each analysis, we adjust the MCMC iterations such that the chains with different transition kernels run for approximately the same wall time. Specifically, we run the MPXV experiment for around 3 hours where the MCMC with HMC runs for 15,000 iterations and the MCMC with ‘univariate’ kernel runs for 1,000,000 iterations. We run the BRCA1 experiment for around 10 hours where the MCMC with HMC runs for 20,000 iterations and the MCMC with ‘univariate’ kernel runs for 600,000 iterations. In the large MPXV example, the univariate kernel achieves 0.0095 ESS/min for the dimension with the lowest effective sample size, while the HMC kernel achieves 3.41 ESS/min, corresponding to a relative speedup of approximately 359.6-fold. In the small BRCA1 example, the univariate kernel achieves 1.51 ESS/min for the dimension with the lowest effective sample size, while the HMC kernel achieves 1.86 ESS/min, corresponding to a relative speedup of approximately 1.23-fold. We perform all benchmarking experiments utilizing 16 CPU threads for BEAGLE calculations (with SSE for the MPXV example and without SSE for the BRCA1 example) on a Super Micro SYS-740GP-TNRT system with two Intel Xeon Silver 4214 CPUs and 512 Gb memory. Figure 4 illustrates the branch-specific substitution parameter estimates binned by the ESS per second for each dimension for the two data examples. The efficiency of HMC is overwhelmingly better in the MPXV example, but only slightly better compared to the univariate sampler in the BRCA1 example largely due to the considerable difference in data set size.

## 5 Discussion

We presented a new algorithm for calculating the gradient of the phylogenetic model likelihood w.r.t. branch-specific substitution process parameters. Our approach achieves linear complexity in the number of sequences by extending the post-order traversal in Felsenstein’s pruning algorithm and our previous approach that calculates the gradient w.r.t. the branch lengths (Felsenstein, 1973, 1981; Ji et al., 2020). The new algorithm enables the application of gradient-based methods for optimization such as the BFGS and sampling such as HMC on learning these high-dimensional branch-specific substitution parameters. Interestingly, previous work achieved impressive progress using fast approximations of such gradients with bounded error (Magee et al., 2024; Didier et al., 2024) on sampling using HMC. While our new algorithm provides exact analytic gradient calculations with a computational complexity of *O*(*Nm*^3^) compared to the *O*(*Nm*^2^) complexity for the approximate gradient algorithms, the efficiency trade-off of the numerical error and computational burden for the Hamiltonian dynamic integration that in-return affect HMC’s sampling efficiency remains to be investigated. A direct and additional application of our new exact gradient algorithm is for optimization that typically involves obtaining MLEs, maximum a posterior estimates and variational analyses.

In Ji et al. (2020), we introduced a linear-time algorithm that calculates the likelihood and its gradient w.r.t. all branch lengths through the post-order and the complementary pre-order traversal. In this work, we presented another linear-time algorithm that calculates the gradient w.r.t. all branch-specific substitution parameters through the same pre-order traversal. In fact, the post- and pre-order partial likelihood vectors (i.e., $p_{i}$ and $q_{i}$) are exactly the same in both cases and therefore only need one single update. The difference between the two gradient calculations is in the final reduction in the numerator of Equation 11 and Equation 13 where the sandwich calculation utilizes the differential matrix and the post- and pre-order partial likelihood vectors. Because of this, one can calculate multiple *O*(*N*)-dimensional gradients (for different substitution parameters and branch lengths) in one single pre-order traversal in practice. For derivative w.r.t. branch length, the differential matrix (i.e., the middle matrix in the sandwich) is simply the instantaneous transition matrix $Q_{i}$ where $Q_{i}$ is independent from the branch length. However, when calculating the derivative w.r.t. parameter within the generator matrix $Q_{i}$, one needs to construct the directional derivative of the matrix exponential as in Najfeld and Havel (1995). To match the sandwich calculation as in Ji et al. (2020) and make the derivative of the pre-order partial likelihood vector the same operation as left-multiplying another matrix to itself so that one re-uses the pre-order partial likelihood vectors, we need to right-multiply the inverse of the matrix exponential (i.e., the transition probability matrix) to the directional derivative matrix. This seemingly additional calculation actually results in reduced scalar exponential calculations in the construction of the middle matrix in the sandwich calculation (e.g., compare Equation 10 with Equation 8).

Our gradient algorithm requires the transition probability matrices and their eigen decomposition being readily available after the likelihood calculation through post-order traversals (e.g., as in Equation 11 and Equation 13). Since we compute the transition probability matrices using eigen decomposition—which has a computational cost of *O*(*m*^3^), where *m* is the size of the state space and *m* × *m* is the dimension of the matrix—and because each branch-specific substitution parameter requires a separate decomposition, the total computational cost (including both eigen decomposition and matrix exponentiation) becomes significant, especially for large state spaces. This is the case for the BRCA1 data example where m=61 and N=8 and this may explain why HMC’s performance is less impressive compared to the MPXV example where m=4 and N=138. Fortunately, these large state space models usually lead to sparse instantaneous transition matrices (i.e., $Q_{i}$ is sparse), allowing one to update the post- and pre-order partial likelihood vectors via the matrix action calculations that significantly reduce the computational cost from cubic $O\left(m^{3}\right)$ down to near quadratic $O\left(fm^{2}\right)$ depending on the sparsity f of $Q_{i}$ (Al-Mohy and Higham, 2011; Ji et al., 2016; Sherlock, 2021). However, the proposed gradient algorithm will not be readily applicable for these matrix action calculations because neither the transition probability matrices nor their eigen decompositions are available such that novel algorithms need to be developed to accommodate these promising techniques.

A caveat of our MLE optimization comparison is that it does not include other widely used optimization criteria. For example, the “virtual root” method employed by several popular phylogenetic software packages such as PhyML (Boussau and Gouy, 2006; Guindon et al., 2010) and RAxML (Stamatakis, 2014). The central finite difference numerical method has a computational complexity of *O*(*N*^2^) and over-estimates the cost for computing gradient numerically. The virtual root method, on the other hand, achieves a lower computational complexity of *O*(*N*) by relocating the root adjacent to the branch being altered. By systematically moving the root to each branch in the tree, only one partial likelihood vector needs to be recomputed at a time (effectively constructing the pre-order partial likelihood vectors). When such vectors are available (e.g., via the virtual root traversal), our analytic gradient algorithm achieves comparable computational cost while offering higher accuracy. Another caveat of our MLE optimization comparison is that there lacks a thorough investigation on external factors that may influence run time. For instance, variations in run time may stem from differences in starting parameter values, computing hardware structures (e.g., x86 vs. ARM), operating systems, or even compiler optimization flags (such as “-O2” versus “-O3”). Although randomizing the starting point could help isolate such influences, this is beyond the scope of the current work. Instead, our comparison focuses on per iteration performance, which more directly reflects differences in algorithmic efficiency. Interestingly, full-run comparisons from our limited experiments suggest greater numerical instability in the numerical gradient method, as evidenced by a notably lower maximized log-likelihood value for the MPXV example and fewer iterations for the BRCA1 example. This instability likely arises from the combined effects of eigen decomposition and partial likelihood vector updates throughout the tree, and its thorough investigation remains an important avenue for future research.

Branch-specific substitution processes have been a popular choice for modeling evolutionary heterogeneity across lineages and have led to various important studies on detecting positive selection pressures in lineages (Muse and Gaut, 1994; Yang, 1998; Yang and Nielsen, 1998, 2002; Murrell et al., 2013). However, most methods typically rely on a fixed tree topology and either specify *a priori* clusters of branches on a phylogeny to share the same substitution process or discourage using many free parameters when assuming that each branch has a specific substitution process (e.g., PAML currently allows a maximum of 8 branch types with different *dN/dS* ratios; Álvarez-Carretero et al., 2023). We explore a Bayesian phylogenetic inference framework that simultaneously employs branch-specific substitution processes while searching the tree topologies without any *a priori* assumptions on any branches to share the same processes (which is however a special case of our model). The Bayesian bridge shrinkage prior that we place on the increments of branch-specific substitution parameters shows promising potential towards an “automated” change-point solution such that it penalizes the log difference of the substitution parameter of a branch to the parameter of the parent branch while its heavy-tail allows actual signals to pass through. Except for its heavier tails, the Bayesian bridge shrinkage prior works similarly to the normal priors employed in the auto-correlated branch rate models (Thorne et al., 1998) and its counterpart in maximum likelihood methods known as a ‘penalty’ term. The combination of branch-specific substitution parameters and Bayesian bridge shrinkage priors reveals clear change-points in the mutational and selection pressure dynamics in the MPXV and BRCA1 examples respectively without *a priori* clustering branches into discrete levels. While incorporating Markov-modulated modeling (Baele et al., 2021) across sites could potentially extend our approach to account for both branch- and site-specific heterogeneity, the potential identifiability issues and their resolution remain important avenues for future research.

## Figures and Tables

**Table 1:** Maximum likelihood estimate (MLE) inference efficiency using two optimization methods: our proposed gradient method (Analytic) and a central finite difference numerical scheme (Numerical). For each example and method, we report the maximized log likelihood, total time to complete MLE inference, as well as the number of iterations required for optimization. Our proposed method yields a higher maximized log likelihood value with at least 4-fold increase in performance per iteration across the entire inference.

		Analytic			Numerical			Speedup	
			
Example	*N*	lnL	Time(s)	Iterations	lnL	Time(s)	Iterations	per Iteration	Total

MPXV	138	−272882.9251	7.0	244	−272889.8235	1900.5	737	89.9×	271.7×
BRCA1	8	−9358.35647	52.5	223	−9358.35651	74.9	74	4.3×	1.4×
